# hnRNP F: An emerging orchestrator of RNA processing and gene expression

**DOI:** 10.1016/j.isci.2026.117417

**Published:** 2026-09-08

**Authors:** Zhuanhuai Wang, Hengjun Huang, Hui Li

**Affiliations:** 1 School of Pharmacy, Jiangxi University of Chinese Medicine, Nanchang 330004, China; 2 Jiangxi Province Key Laboratory of Traditional Chinese Medicine Pharmacology, Institute of Traditional Chinese Medicine Health Industry, China Academy of Chinese Medical Sciences, Nanchang 330115, China; 3 Jiangxi Health Industry Institute of Traditional Chinese Medicine, Nanchang 330115, China; 4 Institute of Chinese Materia Medica, China Academy of Chinese Medical Sciences, Beijing 100700, China

**Keywords:** hnRNP F, alternative splicing, RNA processing

## Abstract

Heterogeneous nuclear ribonucleoprotein F (hnRNP F), a member of the hnRNP family, is an essential regulator of RNA metabolism, influencing splicing, translation, stability, and telomere maintenance. This review provides a comprehensive overview of the regulation of expression and multifunctional roles of hnRNP F. Owing to the high degree of sequence homology and functional similarity between hnRNP F and hnRNP H/H′, this review also integrates discussions on hnRNP H/H′ to facilitate a comparative and holistic understanding of their biological significance.

## Graphical abstract

**Figure:**
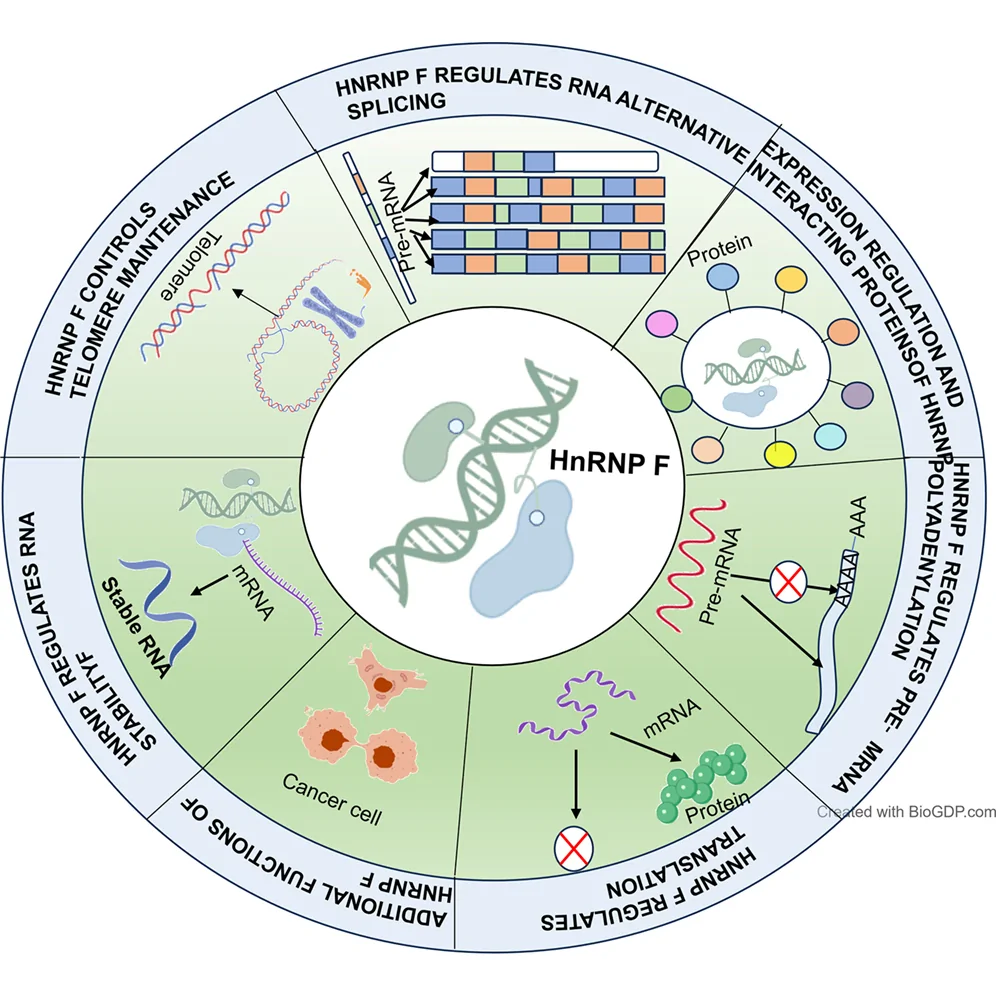

## Teaser

Molecular biology; Molecular mechanism of gene regulation

## Introduction

The heterogeneous nuclear ribonucleoprotein (hnRNP) family comprises a major class of ribonucleic acid (RNA)-binding proteins. With the exception of hnRNP U, all major members harbor one or more RNA-binding domains—including canonical RNA recognition motifs (RRMs), K-homology (KH) domains, or Arginine-Glycine-Glycine (RGG) repeats—whereas the hnRNP F/H subfamily is distinguished by its three characteristic quasi-RNA recognition motifs (qRRM) domains.^1^ The number, combination, and sequential arrangement of these domains vary considerably among family members, providing the molecular basis for their functional specificity. For instance, hnRNP C harbors a single RRM and preferentially binds U-rich sequences,^2^^,^^3^ whereas hnRNP L possesses four RRMs and binds to C/A-rich sequences,^4^ and hnRNP I (PTBP1) recognizes pyrimidine-rich sequences.^5^ In contrast, the hnRNP F/H subfamily is distinguished by its three characteristic qRRMs, which confer specific recognition of G-rich (GGG) sequences, demonstrating a unique RNA-binding property among the family members.^3^^,^^6^

**Figure 1:**
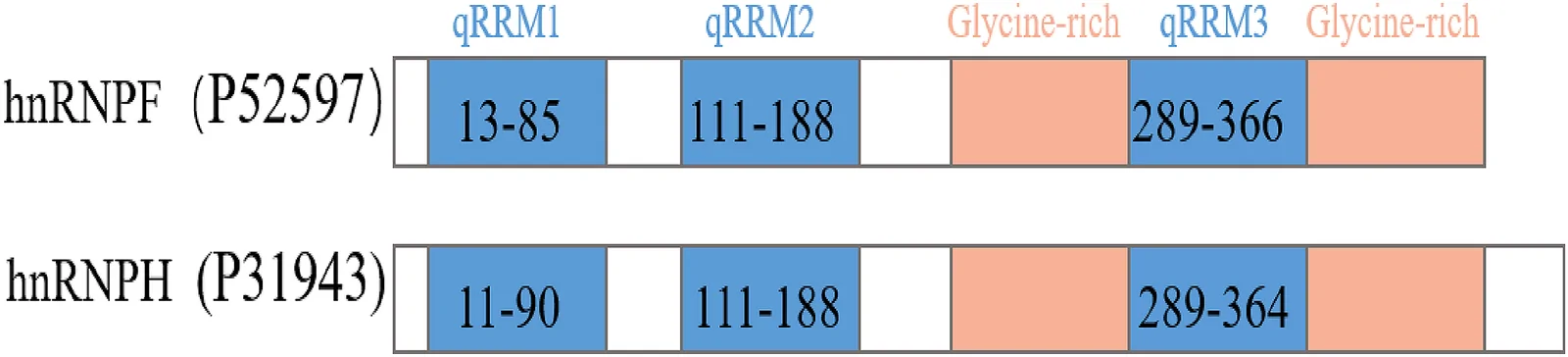
Domain architecture of human hnRNP F and hnRNP H proteins Domain architectures of human hnRNP F (Uniprot: P52597) and hnRNP H (Uniprot: P31943), constructed based on their amino acid sequences, with putative functional domains indicated. The first two qRRM domains of hnRNP F and hnRNP H share 94% structural similarity.

Heterogeneous nuclear ribonucleoprotein F (hnRNP F) and the closely related heterogeneous nuclear ribonucleoprotein H/H′ (hnRNP H/H′) were initially identified via UV-crosslinking and immunopurification techniques.^1^ These proteins share high sequence homology, a common epitope, and a characteristic domain architecture comprising three qRRMs, with two glycine-rich domains flanking qRRM3 (Figure 1).^6^ Specifically, full-length human hnRNP F, H, and H′ share 40% sequence identity, whereas the first two qRRM domains exhibit a sequence identity as high as 77%, with structural similarity reaching 94%.^7^ Furthermore, monoclonal antibodies produced against bacterially expressed hnRNP F were specific for both hnRNP F and hnRNP H, indicating that the two proteins are highly related immunologically and share identical peptides.^8^ Early work by Swanson and Dreyfuss first demonstrated that hnRNP F/H binds to poly(G) RNA,^3^ while the glycine-rich domains mediate protein-protein interactions, notably with other hnRNPs. Specifically, the N-terminal glycine-rich domain facilitates nuclear import through its interaction with transportin 1 (TRN1).^9^ Subsequent *in vitro* studies by Caputi and Zahler identified the GGGA motif as the core consensus sequence recognized by hnRNP F/H.^3^^,^^10^ Subsequent structural analyses have elucidated the molecular basis for this recognition. Nuclear magnetic resonance (NMR) spectroscopy revealed the solution-state structure of the three qRRMs in hnRNP F, showing a spatial arrangement similar to canonical RRMs but with unique secondary structures at the N terminus of qRRM1.^7^ This structural insight is supported by molecular dynamics simulations, which indicate that qRRM2 binds with high affinity to G-tract RNA, an interaction severely compromised by mutations in the RNA sequence.^11^ Further NMR studies demonstrated that qRRM3 specifically binds single-stranded G-rich sequences (ssRNA), thereby preventing the formation of G-quadruplex (G4) structures.^12^ Ultimately, by binding to contiguous G-tracts, hnRNP F can remodel RNA structure to modulate pre-messenger RNA (mRNA) splicing.^13^

Distinct subcellular localization and tissue expression profiles underscore functional differences between hnRNP F and hnRNP H/H′. Immunofluorescence analyses consistently show that hnRNP H/H′ is predominantly nuclear across various cell types.^14^ In contrast, hnRNP F exhibits a dual distribution in both the nucleus and cytoplasm, suggesting additional cytoplasmic functions. Regarding tissue expression, hnRNP F is ubiquitously expressed, with notably high levels in the prostate. hnRNP H/H′ expression, however, is relatively low in several tissues, including the liver, pancreatic exocrine glands, thyroid, and heart. These differential patterns extend to cancer contexts. Generally, hnRNP F is widely detected in both compartments of tumor cells, whereas hnRNP H/H′ remains primarily nuclear, with higher nuclear than cytoplasmic levels. Notably, hnRNP H/H′ is frequently upregulated in cancers of tissues where its basal expression is normally low (e.g., pancreatic, hepatocellular, and gastric cancers). hnRNP F, by contrast, shows context-dependent regulation, being downregulated in hepatocellular carcinoma but upregulated in gastric cancer.^15^ Collectively, despite their high sequence homology and shared recognition of G-rich RNA motifs, hnRNP F and hnRNP H/H′ are regulated by distinct mechanisms and exhibit unique, often divergent, expression patterns across tissues and cell types, hinting at non-overlapping biological roles.

### Expression regulation and interacting proteins of hnRNP F

The expression of hnRNP F is regulated by a diverse array of factors, including RNA-binding proteins, non-coding RNAs, and hormones. For instance, the absence of specific RNA-binding protein variants, such as the selective RNA-binding protein quaking I (QKI), leads to aberrant splicing events in murine oligodendrocytes. The cytoplasmic isoform QKI-6 can suppress the expression of both hnRNP F and hnRNP H proteins, thereby rescuing these abnormal splicing patterns. Intriguingly, while QKI-6 directly binds to hnRNP H mRNA to repress its translation, it does not interact with hnRNP F mRNA, suggesting an indirect mechanism for hnRNP F regulation.^16^ Beyond RNA-binding proteins, non-coding RNAs are also key regulators. In glioma cells, microRNA-128 (miR-128) was identified as a direct upstream regulator that inhibits hnRNP F expression, as determined by pulsed stable isotope labeling with amino acids in cell culture.^17^ Similarly, in thyroid cancer, human microRNA-139-5p (hsa-miR-139-5p) suppresses *HNRNPF* expression by directly targeting its 3′ untranslated region (3′ UTR), leading to reduced hnRNP F protein levels that have been associated with altered splicing of genes within the RTK/RAS/MAPK and PI3K/AKT/mTOR signaling pathways.^18^ Furthermore, studies manipulating long non-coding RNA (lncRNA) metastasis associated lung adenocarcinoma transcript 1 (*MALAT1*) levels indicate that hnRNP F expression is also subject to regulation by lncRNA.^19^ Hormonal regulation is exemplified by insulin, which not only upregulates hnRNP F and hnRNP K expression *in vitro* via the p44/42 MAPK signal pathway but also exerts a similar effect in the kidney tissues of Akita diabetic mice. This upregulation suppresses angiotensinogen (*Agt*) gene transcription and attenuates renal damage, suggesting that hnRNP F may serve as a potential therapeutic target for diabetes.^20^

The diverse functions of hnRNP F are also determined by its interactions with various binding partners. Early *in vitro* studies demonstrated that hnRNP F interacts with cap-binding protein 80 (CBP80) and cap-binding protein 20 (CBP20) in an RNA-independent manner, although RNA presence can stabilize this interaction.^21^ The reduced pre-mRNA intron removal efficiency observed in hnRNP F-depleted HeLa cell nuclear extracts, together with the finding that hnRNP F can directly interact with the CBP80/CBP20 components of the nuclear cap-binding complex (CBC) and is preferentially recruited to CBC-bound RNA, collectively suggest that hnRNP F may participate in the regulation of pre-mRNA intron removal through its interaction with the CBC.^21^ Further evidence implicates hnRNP F in splicing regulation through its association with Dim1, a component of the U4/U6·triple small nuclear ribonucleoprotein (U4/U6 tri-snRNP). U5 tri-snRNP, as identified by yeast two-hybrid screening.^22^ The functional significance of these interactions is cell context dependent. For instance, in prostate cancer, hnRNP F recruitment to splice sites depends on its interaction with the histone demethylase (JMJD1A).^23^ In T cells, hnRNP F binds the exon 2 region of Forkhead box P3 (*FOXP3*) pre-mRNA to regulate its splicing.^24^ Beyond splicing, hnRNP F mediates alternative splicing of the Yes-associated protein (YAP) 3′ UTR in cancer cells to repress *YAP* expression,^25^ and directly interacts with targeting protein for Xklp2 (*TPX2*) to promote proliferation in bladder cancer.^26^ Non-coding RNAs can also modulate hnRNP F activity, as exemplified by cytoplasmic *circCacna1c*, which sequesters hnRNP F to inhibit its nuclear translocation and subsequent receptor-interacting serine/threonine kinase 1 (*RIPK1*) expression, ultimately improving cardiac function.^27^ Furthermore, under high-glucose conditions, upregulation of hnRNP F promotes its binding to G-rich RNA sequences, recruiting cooperating splicing factors to alter exon inclusion in transcripts of tumor necrosis factor alpha (TNF-α)/nuclear factor kappa B (NF-κB) pathway genes and suppress inflammation.^28^ Collectively, these studies reveal that hnRNP F’s function depends not only on its RNA-binding domains but also critically on its dynamic protein-protein interactions.

### hnRNP F regulates RNA alternative splicing

hnRNP F is a key regulator of a plethora of RNA alternative splicing events. Early studies established its role in general mRNA splicing, and subsequent research has extensively documented its function in promoting exon skipping or inclusion, thereby generating protein isoforms with distinct or even opposing functions.

hnRNP F regulates the RNA alternative splicing of numerous crucial genes in normal cells by binding to G-rich elements. For instance, in neuronal cells, hnRNP F serves as a component of a neuron-specific splicing regulatory complex that binds to the downstream control sequence (DCS) of the cellular sarcoma (*c-src*) pre-mRNA N1 exon, where it cooperates with a neuron-specific 75 kDa protein and other constitutive proteins to promote the inclusion of the N1 exon.^29^^,^^30^ Its role, often in conjunction with the homologous proteins hnRNP H/H′, extends to various cellular contexts. In oligodendrocytes, hnRNP F and H/H′ interact with U1 small nuclear ribonucleoprotein (U1 snRNP) to promote the selection of the DM20 isoform over myelin proteolipid protein (PLP) during the splicing of *PLP* mRNA, with hnRNP H playing the dominant role and hnRNP F exerting a synergistic effect.^31^^,^^32^ Furthermore, they compete with alternative splicing factor/splicing factor 2 (ASF/SF2) for binding to exon IIIc of fibroblast growth factor receptor 2 (*FGFR2*), thereby promoting exon skipping.^33^ Additional splicing targets include TNF receptor superfamily member 5 (*Cd40*) in B cells, where hnRNP F binding to a G-tract promotes exon 6 inclusion;^34^ the *HRAS* proto-oncogene, where hnRNP F/H facilitates exon 5 inclusion by interacting with downstream G elements^35^; and the insulin receptor (*INSR*), where hnRNP F promotes exon 11 inclusion by recognizing an intronic AGGGA sequence in a process involving SRSF1 and antagonized by hnRNP A1.^36^ Finally, in human embryonic stem cells (*hESCs*), high expression of hnRNP F and H/H′ modulates the splicing of T cell factor 3 (*TCF3*), favoring the E12 variant, while a switch to the E47 variant occurs during differentiation.^37^

hnRNP F plays a critical, yet, context-dependent role in tumorigenesis by regulating the alternative splicing of key oncogenes and tumor suppressors. For example, hnRNP F binds to a G-rich element (B2G) on *Bcl-x* pre-mRNA, shifting the splicing balance toward the pro-apoptotic isoform B cell lymphoma-extra short isoform (Bcl-xS) and promoting apoptosis in HeLa cells.^38^ Similarly, hnRNP F has been implicated in promoting the skipping of *ENOX2* exon 4 through binding to a GGGA ESS motif within the *ENOX2* pre-mRNA. However, the evidence supporting this interaction is entirely indirect: the study depended solely on mini-gene constructs, site-directed mutagenesis, and computational predictions, and critically lacked direct binding assays (e.g., EMSA, RIP, or RNA pull-down) to verify hnRNP F engagement with the *ENOX2* transcript. Additionally, the potential involvement of hnRNP H/H′ in this splicing event was not evaluated, leaving the possibility of redundant or cooperative regulation unexamined.^39^ Conversely, hnRNP F can also exert anti-apoptotic effects; its knockdown, along with hnRNP K and H/H′, increases the pro-apoptotic isoform Mcl-1S, indicating a role in sustaining the anti-apoptotic myeloid cell leukemia-1 long (Mcl-1L) isoform.^40^ HnRNP F functions as an oncogenic facilitator in prostate cancer, as evidenced by studies demonstrating that its knockdown suppresses production of the androgen receptor splice variant 7 (*AR*-V7), a well-established driver of therapy resistance. Given that this study was predominantly performed in three prostate cancer cell lines (CWR22Rv1, LN95, and VCaP), the generalizability of its findings to other prostate cancer subtypes or primary patient-derived cells warrants further investigation.^23^ In esophageal squamous cell carcinoma, hnRNP F interacts with lncRNA-UCA1 to promote a pro-oncogenic splicing switch in *FGFR2*. Nevertheless, whether hnRNP F directly recognizes specific *cis*-elements on the *FGFR2* pre-mRNA or functions indirectly via recruiting additional splicing cofactors remains to be determined.^41^ The regulatory mechanism of hnRNP F involves recognition of specific RNA sequences and structures. A G4-dependent splicing regulation of *CD44* has been identified as a mechanism by which hnRNP F inhibits epithelial-mesenchymal transition (EMT) and acts as a tumor suppressor in breast cancer.^42^ In summary, hnRNP F can function as either a tumor suppressor or a promoter, with its effect being determined by the specific cellular context and target genes.

hnRNP F may be deceived in some cases, resulting in incorrect alternative splicing events. In some diseases, mutations in intronic sequences create pseudoexons. hnRNP F recognizes G-rich sequences within these pseudoexons and promotes their aberrant inclusion, leading to gene expression dysregulation.^43^ Such erroneous alternative splicing events may represent a potential pathogenic mechanism underlying many cancers and other diseases.

## hnRNP F controls telomere maintenance

Telomeres are complexes of repeated non-coding DNA sequences and proteins located at the ends of chromosomes. In recent years, studies have shown that their length is closely related to aging.^44^ Telomeric repeat-containing RNA (TERRA) is a long non-coding RNA transcribed from telomeric DNA and contains G-rich UUAGGG repeats. Studies have shown that hnRNP F can bind to TERRA transcripts, alter their localization on telomeres, and regulate TERRA abundance and telomere length. In this study, knockdown of hnRNP F led to increased TERRA levels, suggesting that hnRNP F functions as a negative regulator of TERRA abundance.^45^ In a more recent study, hnRNP F and hnRNP H/H′ were found to simultaneously bind to the 5′ region of the human telomerase RNA component (*hTERC*) and the telomerase holoenzyme, thereby maintaining telomerase activity and telomere length. In that context, knockout or overexpression of hnRNP F in tumors and stem cells demonstrated that hnRNP F promotes proliferation and delays senescence.^46^ Notably, these two studies describe distinct regulatory mechanisms: for the study by Silanes et al., it was found that hnRNP F directly binds to the UUAGGG repeats of TERRA through its qRRM domains. Knockdown of hnRNP F significantly extended the half-life of TERRA from approximately 5.7 to over 8 h, indicating that hnRNP F reduces TERRA steady-state levels by accelerating its degradation. Therefore, depletion of hnRNP F may lead to increased TERRA accumulation at telomeres. In contrast, Xu et al. proposed that the underlying mechanisms may involve hnRNP F/H binding to the 5′ end of *hTERC* to prevent G4 formation and ensure correct P1 helical structure, thereby guaranteeing proper telomere synthesis. Alternatively, hnRNP F/H may serve as components of the telomerase RNP complex, facilitating its correct assembly or localization to telomeres. Consequently, knockdown of hnRNP F/H results in decreased telomerase activity, which in turn leads to telomere shortening.

## hnRNP F regulates pre-mRNA polyadenylation

In B cells, high hnRNP F expression competitively inhibits cleavage stimulation factor-64 (CstF-64) binding to the GU-rich element downstream of the proximal secretory poly(A) site (sec-pA), thereby suppressing sec-pA use and favoring the distal membrane poly(A) site (mb-pA) to produce the longer mRNA encoding membrane-bound immunoglobulin (Ig). In plasma cells, reduced hnRNP F expression relieves this inhibition specifically at the sec-pA site, allowing production of the shorter mRNA encoding secreted Ig.^47^ In another study, it was also proved that hnRNP F inhibits the binding of CstF-64 to the pre-mRNA substrate of SV40 (simian virus 40) containing guanine-rich sequence (GRS), but hnRNP H2 does not have inhibitory function.^48^ Another study demonstrated that hnRNP F can also affect mRNA 3′ end processing through a distinct mechanism. Under DNA damage conditions, mRNA 3′ end processing is repressed. However, the p53 pre-mRNA can specifically recruit hnRNP F/H through the G4 structure formed downstream of its cleavage site. This binding promotes the recruitment of CstF to the p53 polyadenylation signal, thereby ensuring proper 3′ end processing and polyadenylation efficiency of p53 pre-mRNA, which is critical for the generation of mature p53 mRNA and the subsequent p53-mediated apoptotic response.^49^ It should be noted that in B cells, hnRNP F binds to the linear GU-rich sequence downstream of the sec-pA site in Ig pre-mRNA, whereas in DNA-damaged cells, hnRNP F/H binds to the G4 structure downstream of the cleavage site in p53 pre-mRNA. The differences in both sequence composition and secondary structure between these two RNA substrates as well as the distinct proteins that may participate under different conditions, lead hnRNP F to exert its regulatory effects through two distinct mechanisms—competitively inhibiting CstF-64 binding or recruiting CstF to the poly(A) signal—thereby functioning as a negative or positive regulator, respectively. These studies indicated that hnRNP F plays a vital regulatory role in the 3′ end processing of pre-mRNA.

## hnRNP F regulates RNA stability

Beyond its role in splicing, hnRNP F is implicated in mRNA stability regulation, albeit with seemingly paradoxical outcomes. On one hand, hnRNP F facilitates mRNA degradation by interacting with zinc-finger proteins like tristetraprolin (TTP) and its family members butyrate response factor 1 (BRF1) and 2 (BRF2), which bind to the 3′ UTR of target transcripts.^50^ Conversely, other studies demonstrate a stabilizing function for hnRNP F. For instance, it can directly bind to the 3′ UTR of *Snail1* mRNA to inhibit its degradation,^42^ and similarly prolong the half-life of *APP* mRNA in neuronal cells.^51^ Furthermore, hnRNP F can influence mRNA stability indirectly by promoting alternative splicing of the 3′ UTR, as seen with *YAP* mRNA, where a splicing event yields an isoform with reduced half-life.^25^ These contrasting findings highlight the context-dependent nature of hnRNP F in mRNA stability control, suggesting that its effect—stabilization or destabilization—may be determined by cell type, specific mRNA targets, and co-factors. Resolving this duality requires further mechanistic investigation.

## hnRNP F regulates translation

Evidence suggests that hnRNP F may function as a suppressor of mRNA translation. This is supported by studies on its homolog in *Drosophila*, Glorund (Glo), which shares homology with hnRNP F and hnRNP H/H′. Glo binds to the UA-rich motif in the 3′ UTR of *nanos* mRNA—a gene critical for embryonic development—and inhibits its translation.^52^ Intriguingly, while Glo regulates the translation of *nanos* mRNA via UA-rich motifs, it controls the alternative splicing of other RNA targets through G-tract motifs. Further research revealed that Glo possesses two distinct RNA-binding surfaces, enabling it to recognize ssRNA G-rich sequences and double-stranded UA-rich motifs.^53^^,^^54^ This mechanistic insight helps explain the functional diversity of hnRNP F and hnRNP H/H′ in regulating different aspects of RNA metabolism.

Converging evidence indicates that hnRNP F and hnRNP H/H′ collaboratively regulate translation, primarily through their interaction with RNA G-quadruplexes (rG4s). For instance, one study in mice demonstrated that both proteins inhibit the translation of opioid receptor mRNA by binding to a specific M4 sequence in its 5′ UTR.^55^ The underlying mechanism involves the stabilization of rG4 structures upon hnRNP F/H binding.^56^ This translational control is a context dependent, as evidenced by a study in glioblastoma showing that the interaction of hnRNP H/F with the RNA helicase DHX36 promotes the binding of hnRNP H/F to unfolded rG4s, thereby stimulating translation.^56^ While these studies often investigate hnRNP F and H/H′ together, the predominant regulator remains unclear. Given their distinct subcellular localization, hnRNP F is hypothesized to be the major contributor to the translation regulation.

## Additional functions of hnRNP F

Pre-mRNA splicing is a co-transcriptional process. Early research implicated the splicing factor hnRNP F in the regulation of transcription initiation. An *in vitro* study demonstrated that hnRNP F directly interacts with the TATA binding protein (TBP), and their tissue expression patterns are highly similar.^57^ Furthermore, a contemporaneous study revealed that hnRNP F binds to the GC-box within the single-stranded SV40 promoter DNA. This DNA-binding activity was mapped not to its qRRM domains but to the central glycine-rich domain.^58^ Given that hnRNP F is also a component of the RNA polymerase II holoenzyme and exhibits a tissue expression profile similar to that of RNA pol II,^58^ these findings collectively suggest that hnRNP F functions beyond its canonical role in RNA processing and may be directly involved in transcriptional regulation.

hnRNP F is also essential for the translocation of RNA granules. For instance, in neuronal systems, hnRNP F, alongside hnRNP A2, is incorporated into transport granules containing myelin basic protein (*MBP*) mRNA. These granules facilitate the delivery of translationally silenced *MBP* mRNA to oligodendrocytic processes. Upon reaching its destination, the src family non-receptor tyrosine kinase Fyn phosphorylates cytoplasmic hnRNP F, leading to the dissociation of the mRNA granules.^59^

Beyond their roles in mRNA regulation, hnRNP F and H/H′ directly bind to the tRF-GG element of tRNA fragments to suppress *U7 snRNA* transcription. Given that tRF-GG participates in generating diverse non-coding RNAs (e.g., snoRNAs, scaRNAs, and snRNAs), this finding implies that hnRNP F and H/H′ may broadly regulate non-coding RNA synthesis.^60^

hnRNP F can also interact with viruses. Upon viral infection, hnRNP F undergoes cytosolic translocation, where its qRRM1 and qRRM2 domains specifically recognize and bind to the GRS of viral RNA. This interaction facilitates the replication of PRRSV2, as evidenced by a significant reduction in viral yield upon hnRNP F inhibition.^61^ Beyond PRRSV2, hnRNP F/H also modulates the life cycle of other viruses, notably HPV16. hnRNP F/H induces the expression of HPV16 late genes and regulates the splicing of HPV16 L1 mRNA, promoting the inclusion of the central exon between SA3358 and SD3632. In contrast, hnRNP A1 exerts the opposite effect on this exon. The tight regulation of HPV16 late *L1* and *L2* capsid protein expression through this splicing switch is thought to facilitate the persistent infection of HPV16.^62^

## Discussion

**Figure 2:**
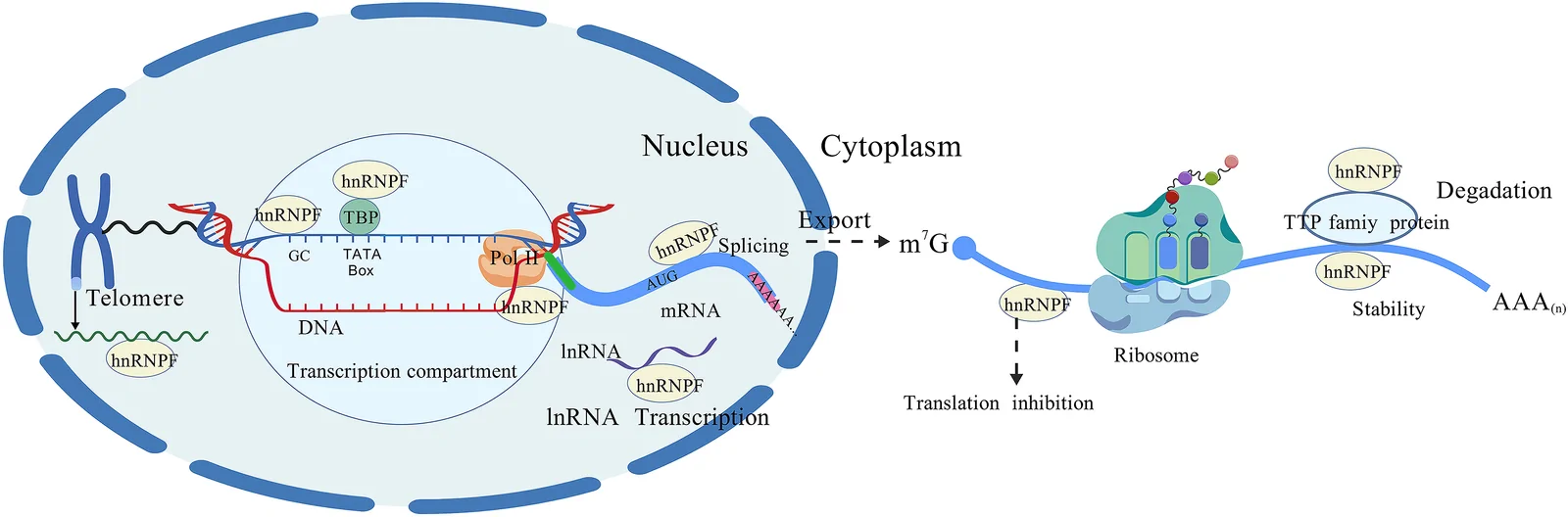
The function of hnRNP F in RNA processing hnRNP F exerts multiple regulatory roles in RNA metabolism within the nucleus and cytoplasm. In the nucleus, hnRNP F contributes to telomere maintenance, transcriptional regulation, long non-coding RNA processing, and mRNA splicing. Following processing, mature mRNA is exported to the cytoplasm. In the cytosol, hnRNP F mediates translational repression and modulates mRNA stability. Created with BioGDP.com.^64^

As a key regulator of RNA metabolism, hnRNP F participates in diverse RNA processing events (Figure 2). Following the recognition of G-rich RNA elements through its qRRM domains, hnRNP F remodels RNA secondary structures—such as stabilizing or unwinding G-quadruplexes—to modulate downstream RNA processing.^63^ In the context of alternative splicing, this structural remodeling changes the accessibility of splicing regulatory elements, and in coordination with splicing factors recruited via the glycine-rich domain, facilitates either exon inclusion or skipping, ultimately giving rise to different mRNA isoforms from a single transcript.^31^^,^^37^^,^^40^ Beyond splicing, hnRNP F plays multifaceted roles: it binds telomeric RNA to maintain telomere integrity, associates with proteins like those in the TTP family to regulate mRNA stability (e.g., by directly binding and stabilizing *Snail* mRNA), and generally acts as a repressor of translation for certain mRNAs. Additionally, emerging evidence suggests its involvement in transcription initiation, RNA particle transport, and non-coding RNA biogenesis. Owing to these diverse functions, hnRNP F is critically implicated in vital processes, such as cell development, differentiation, diabetes pathogenesis, tumorigenesis, and metastasis.

Despite considerable functional overlap between hnRNP F and hnRNP H (Table 1), their distinct intracellular localization and tissue expression patterns suggest unique roles for hnRNP F. HnRNP F predominantly functions in the nucleus to regulate alternative splicing and telomere stability; in the cytoplasm, it may participate in mRNA stability or translational regulation. hnRNP H is also mainly localized to the nucleus, where it primarily regulates alternative splicing, whereas its cytoplasmic functions remain largely unexplored. Importantly, hnRNP F has been shown to mediate the renoprotective effects of insulin in diabetes. Upon insulin stimulation, hnRNP F represses the expression of *Agt* and Bcl2 modifying factor (*Bmf*) while activating the transcription of *Ace*-2, *Sirtuin*-1, and forkhead box O3 alpha (*Foxo3α*), thereby improving diabetic symptoms.^65^^,^^66^^,^^67^^,^^68^ This pathway represents a key mechanism underpinning insulin’s therapeutic action.

**Table 1:** Diverse roles of hnRNP F

Cell types	Targets	Function	Involved hnRNP H	Mechanistic evidence	Reference
Glioblastoma	RG4-containing DDR genes (*Usp1*) DHX36	translation regulation	yes	RNA affinity purification, RP-MS, RT-qPCR, RIP, western blot, and sequential siRNA knockdown	Song et al.^56^
Mouse fibroblasts	telomerase RNA	telomere maintenance	no	biotin pull-down, LC-MALDI MS/MS, RIP, northern blot, and siRNA knockdown	Demanelis et al.^45^
Yeast	interactions with Dim1	pre-mRNA splicing	no	yeast two-hybrid, GST pull-down, and coIP	Zhang et al.^22^
B cell	*IgH* mRNA	polyadenylation sites selection	yes	western blot, UV, IP, EMSA, northern blot, *in vitro* cleavage assay, and stable transfection	Xu et al.^47^
	*Cd40* mRNA	alternative splicing	no	RNA-seq, RT-PCR, RIP, RNA pull-down, and EMSA	Mauger et al.^34^
HeLa cells	*Bcl-x* mRNA	alternative splicing: bcl-xs, bcl-xL	yes	chemical shift perturbation, NMR, and site-directed mutagenesis	Dominguez,^7^ Yamazaki et al.^38^
*Drosophila*	*nanos* mRNA	translation regulation	yes	UV, LC-MS/MS, EMSA, and western blot	Khan et al.^52^
Oligodendrocytes (OLs)	*PLP*/*DM20*	alternative splicing	yes	systematic mutagenesis, LC-MS/MS, western blot, and RT-PCR RNA affinity purification	Min et al.^31^
HEK293T, esophageal squamous cell carcinoma (ESCC) and DT cells	fibroblast growth factor receptor 2 (*FGFR2*) mRNA	alternative splicing: exon skipping	yes	RNAi knockdown, RNA pull-down, coIP, RT-PCR, and ESE finder	Wang and Cambi,^33^ Tyson-Capper and Gautrey^41^
Neuronal cells	pre-mRNA of *c-src*	alternative splicing	no	*In vitro* splicing assay, IP, UV, SDS-PAGE, and SR protein functional analysis	Jiang et al.^29^
HepG2 and HEK293 cells	insulin receptor gene (*INSR*)	alternative splicing: exon inclusion	no	linker scanning mutagenesis, RNA pull-down, MS, RT-PCR, RNA pull-down, and siRNA knockdown	Chen et al.^36^
hESCs	T cell factor 3 (*TCF3*)	alternative splicing: E12 and E47	yes	RNA-seq, MATS, RT-PCR, RNA pull-down, western blot, CLIP-qPCR, and siRNA knockdown	Talukdar et al.^37^
Breast cancer cells	myeloid leukemia 1 (*Mcl-1*)	alternative splicing: exon skipping	yes	RT-PCR, IP, western blot, gel electrophoresis, and siRNA knockdown	Tang et al.^40^
MCF-10A, BT-20	tumor-associated NADH oxidase (*ENOX2*)	alternative splicing	no	site-directed mutagenesis, RT-PCR, western blot, Splicing Rainbow program, and GFP minigene assay	Garneau et al.^39^
Prostate cancer	*AR*-V7	alternative splicing	no	RT-qPCR, RNA pull-down, RIP, and ChIP	Fan et al.^23^
Bladder cancer	*Snail1* mRNA	mRNA stability	no	RIP, truncation and mutant assay, RT-qPCR, and actinomycin D	Wang et al.^42^
Human embryonic stem cell line H9 (WA09)	tRF-GG element of the tRNA fragment (TRF)	InRNA transcription	yes	RNA-seq, RT-qPCR, and northern blot	White et al.^60^

hnRNP F exhibits a paradoxical role in tumorigenesis, functioning as either an oncogene or a tumor suppressor in a context-dependent manner. For instance, it is upregulated in bladder cancer, where it promotes tumor cell proliferation and cell cycle progression by enhancing *TPX2* expression.^26^ Conversely, hnRNP F acts as a tumor suppressor in glioma by arresting the cell cycle. In breast cancer, it inhibits epithelial-mesenchymal transition (EMT) through regulating the alternative splicing of *CD44*.^42^

hnRNP F and hnRNP H share highly homologous qRRM domains and G-tract-binding specificity, providing a molecular basis for functional overlap, but the compensation between the two is not a simple functional interchange. In naive B cells, single knockout of hnRNP F does not elicit an obvious phenotype, suggesting that hnRNP H can effectively compensate for its loss. However, in germinal center B cells, hnRNP F deficiency leads to a significant reduction in cell numbers, indicating that under this context, hnRNP H fails to substitute for hnRNP F function, and thus compensation is abrogated.^34^ The functional relationships among members of the hnRNP family are more complex, including both cooperative regulation patterns and antagonistic regulation patterns. For instance, hnRNP A1 and A2B1 are the two members with the highest expression levels and highly homologous sequences in B cells. Deleting either of these genes alone does not disrupt the splicing of exon 14 of *Ezh2*; only when both genes are simultaneously deleted does the Ezh214 isoform accumulate significantly, accompanied by impaired germinal center B cell response, indicating a cooperative regulatory relationship between the two.^69^ In contrast, hnRNP L and hnRNP LL exert opposing functions: hnRNP L recruits the splicing repressor PTB via its unique proline-rich region (PRR) to promote skipping of the P3A exon, whereas hnRNP LL, which lacks this PRR domain, fails to interact with PTB and consequently favors P3A exon inclusion.^70^ A particularly striking example of antagonistic regulation between hnRNP F and other family members comes from the alternative splicing of *Cd40* in B cells. Although hnRNP A1 and A2B1 promote skipping of *Cd40*exon 6, hnRNP F exerts the opposite effect, favoring exon inclusion. Both pairs of proteins recognize G-tracts for splicing regulation, raising the possibility that they compete for access to the same *cis*-regulatory elements on the *Cd40* pre-mRNA. Therefore, hnRNP family members may act either synergistically to ensure functional robustness or antagonistically to achieve fine-tuned regulation, depending on the specific splicing event and cellular context. Notably, many previous studies investigating hnRNP F function have neither performed direct knockout of the hnRNP F gene nor concurrently examined the contribution of hnRNP H, thereby precluding exclusion of potential compensatory effects exerted by hnRNP H/H′. Consequently, it remains difficult to definitively discern whether the observed phenotypes arise from the independent action of hnRNP F or from the synergistic interplay between hnRNP F and H/H′.

Although considerable progress has been made in hnRNP F/H biology, several critical questions remain to be addressed. First, the molecular mechanism by which hnRNP F and H/H′ achieve selective recognition of target RNAs despite sharing highly homologous qRRM domains remains elusive. While specific antibodies capable of distinguishing hnRNP F from H/H′ already exist, their application in side-by-side CLIP-seq studies remains rare. Future efforts should prioritize parallel, transcriptome-wide profiling of both proteins to unambiguously define the degree of target overlap and paralog-specific binding events. Second, the regulatory role of RNA secondary structure in modulating hnRNP F/H activity warrants further in-depth investigation. The splicing regulatory function of hnRNP F/H is highly dependent on the local structural status of G-tracts: when G-tracts adopt single-stranded or readily unwindable conformations, hnRNP F/H can bind effectively and exert their regulatory functions; however, when G-tracts are stably “locked” into G4 structures, protein binding and functional activity may be significantly attenuated. Third, the extent of functional redundancy between hnRNP F and H/H′, as well as the boundary conditions governing such redundancy, remain to be systematically evaluated. Given the high sequence similarity of their qRRM domains and their co-expression in multiple cell types, the phenotypes observed to date cannot fully exclude the potential compensatory or synergistic contributions of H/H′. Therefore, future investigations should employ double-knockout or conditional-knockout animal models in conjunction with transcriptome-wide RNA-protein interaction profiling technologies such as CLIP-seq or RIP-seq, in order to unequivocally distinguish hnRNP F-specific functions that are independent of H/H′ contribution, thereby providing critical experimental evidence toward resolving the aforementioned outstanding questions.

## Acknowledgments

This study was supported by the 10.13039/501100001809National Natural Science Foundation of China (grant no. 32400739), 10.13039/501100004479Jiangxi Province Natural Science Foundation (grant no. 20242BAB20274), and Fundamental Research Funds for the Central Public Welfare Research Institutes (grant nos. ZZ17-ND-12, ZZ17-YQ-053, JSYL2025002, and PY-2025002B). We thank Prof. Xijun Ou from the 10.13039/501100012449Southern University of Science and Technology (10.13039/501100012449SUSTech, Shenzhen, China) for his constructive suggestions on this manuscript.

## Author contributions

Z.W., H.H., and H.L. wrote this paper. All authors reviewed the manuscript. All authors have read and agreed to the published version of the manuscript.

## Declaration of interests

The authors declare no conflicts of interest.
